# Therapist-Delivered Versus Care Ally–Assisted Massage for Veterans With Chronic Neck Pain: Protocol for a Randomized Controlled Trial

**DOI:** 10.2196/38950

**Published:** 2022-09-27

**Authors:** Niki Munk, Joanne K Daggy, Erica Evans, Matthew Kline, James E Slaven, Brian Laws, Trevor Foote, Marianne S Matthias, Matthew J Bair

**Affiliations:** 1 Department of Health Sciences School of Health and Human Sciences Indiana University Indianapolis, IN United States; 2 Australian Research Centre in Complementary and Integrative Medicine Massage & Myotherapy Australia Fellow and Visiting Faculty of Health University of Technology Sydney Sydney Australia; 3 Department of Biostatistics and Health Data Science School of Medicine Indiana University Indianapolis, IN United States; 4 Richard L. Roudebush Veterans Affairs Medical Center Center for Health Information and Communication Indianapolis, IN United States; 5 School of Medicine Indiana University Indianapolis, IN United States; 6 Regenstrief Institute Indianapolis, IN United States

**Keywords:** Veterans, chronic neck pain, integrative medicine, whole health, modified trial design, therapist-delivered versus care ally–assisted massage for Veterans with chronic neck pain, TOMCATT

## Abstract

**Background:**

Chronic neck pain (CNP) is prevalent, and it reduces functional status and quality of life and is associated with deleterious psychological outcomes in affected individuals. Despite the desirability of massage and its demonstrated effectiveness in CNP treatment, multiple accessibility barriers exist. Caregiver-applied massage has demonstrated feasibility in various populations but has not been examined in Veterans with CNP or compared in parallel to therapist-delivered massage.

**Objective:**

This manuscript described the original study design, lessons learned, and resultant design modifications for the Trial Outcomes for Massage: Care Ally–Assisted Versus Therapist-Treated (TOMCATT) study.

**Methods:**

TOMCATT began as a 3-arm, randomized controlled trial of 2 massage delivery approaches for Veterans with CNP with measures collected at baseline, 1 and 3 months after intervention, and 6 months (follow-up). Arm I, care ally–assisted massage, consisted of an in-person, 3.5-hour training workshop, an instructional DVD, a printed treatment manual, and three 30-minute at-home care ally–assisted massage sessions weekly for 3 months. Arm II, therapist-treated massage, consisted of two 60-minute sessions tailored to individual pain experiences and treatments per week for 3 months. The treatments followed a standardized Swedish massage approach. Arm III consisted of wait-list control.

**Results:**

Retention and engagement challenges in the first 30 months were significant in the care ally–assisted massage study arm (63% attrition between randomization and treatment initiation) and prompted modification to a 2-arm trial, that is, removing arm I.

**Conclusions:**

The modified TOMCATT study successfully launched and exceeded recruitment goals 2.5 months before the necessary COVID-19 pause and is expected to be completed by early 2023.

**Trial Registration:**

ClinicalTrials.gov NCT03100539; https://clinicaltrials.gov/ct2/show/NCT03100539

**International Registered Report Identifier (IRRID):**

DERR1-10.2196/38950

## Introduction

### Background

Neck pain is common in adults aged >50 years and is the fourth leading cause of disability in the United States [1]. Chronic neck pain (CNP) reduces functional status and quality of life and is associated with deleterious psychological outcomes in affected individuals. CNP accounts for more than 10 million ambulatory medical visits per year [2].

Medications are most commonly used to treat CNP in clinical practice; however, systematic reviews find limited evidence for effective treatments relative to low back pain, and current therapies show only modest effect sizes [3]. Because medications and other conventional treatments often fail to substantially relieve pain, patients frequently seek other treatments.

In all, 58% of the older adults surveyed used some type of alternative treatment [4], and pain is the primary reason individuals use complementary treatments [5]. After low back pain, CNP is the most common pain condition for complementary health use [6]. Massage is the second (after chiropractic) most commonly used complementary treatment for CNP [7]. Studies have shown that massage is safe, with few risks and rare serious adverse effects [8,9].

Despite its safety and potential benefits, the expense associated with massage therapy limits its accessibility. Teaching informal care allies to provide massages has the potential to improve accessibility. Kozak et al [10] demonstrated the feasibility of caregiver-delivered massage, which led to significant decreases in pain, stress or anxiety, and fatigue in Veterans with cancer. Collinge et al [11] recruited 97 patient or caregiver dyads to practice massage for individuals experiencing cancer. Caregiver-applied interventions led to decreases in patients’ pain, depression, and other cancer-related symptoms [11]. Although the feasibility and benefit of the care ally–applied massage approach have been established in cancer populations, it has not been examined specifically in non–cancer-related musculoskeletal pain populations.

The Trial Outcomes for Massage: Care Ally–Assisted Versus Therapist-Treated (TOMCATT) study began as a 3-arm, randomized controlled trial of 2 massage therapy delivery approaches for CNP. The primary aim of this study was to compare care ally–assisted massage (CA-M) and therapist-treated massage (TT-M) with a wait-list control group (WL-C) for pain-related disability. The secondary outcomes included pain severity, health-related quality of life, depression, anxiety, and stress. Before pausing the TOMCATT activities due to the COVID-19 pandemic, the study design was modified to a 2-arm study. The CA-M arm was discontinued owing to recruitment and adherence challenges.

### Objectives

The primary purpose of this manuscript is to describe the original TOMCATT study design, the challenges faced during the study’s first 2 years of recruitment, and the resultant design modification to a 2-arm study. Specifically, we describe the original TOMCATT methodology, materials, and procedures in the Methods section and report the initial enrollment and intervention initiation outcomes for the first 30 recruitment months, rationale for the modified study design, and subsequent modified TOMCATT methodology in the Results section. TOMCATT is an active study that has restarted from the COVID-19–related pause and is projected to be concluded by early 2023.

## Methods

### Overall Original Design

TOMCATT’s study population consisted of Veterans with CNP randomized to one of 3 study arms: CA-M, TT-M, or WL-C. All eligible patients had an identified care ally, but only those randomized to the CA-M arm ultimately participated in TOMCATT with their care ally. Those randomized to the CA-M arm attended a group training workshop that taught them a massage routine. After the training, CA-M dyads were asked to complete the learned 30-minute massage routine 3 times per week for 12 weeks and document the massages that were delivered in a log. The TT-M intervention consists of twice weekly, hour-long massage sessions provided by massage therapists trained to deliver semistandardized, individualized treatments.

TOMCATT outcome assessments were conducted via interviews at baseline and 1, 3, and 6 months in all study arms. The primary outcomes were neck pain and related disabilities. The secondary outcomes were other pain measures, health-related quality of life, depression, anxiety, and pain cognition.

### Ethics Approval

TOMCATT was reviewed and approved by the Indiana University Institutional Review Board (#1604689005_9034) and Veterans Administration Research Review Committee (VA project ID:IIR 15-333) and registered with ClinicalTrials.gov (NCT03100539). All participants engaged in a one-on-one informed consent process with the study personnel and provided written informed consent.

### Eligibility

Veterans are eligible if they meet criteria outlined in Textbox 1.

**Trial Outcomes for Massage: Care Ally–Assisted Versus Therapist-Treated study eligibility criteria.**

**Inclusion criteria**

- Aged at least 18 years
- Chronic neck pain for ≥6 months
- At least moderate disability per the Neck Disability Index (score of ≥10)
- Access to a working telephone
- Ability and willingness to attend 2 treatments per week for 12 weeks
- Have a care ally (spouse, partner, family member, or friend) willing to learn and provide message therapy during the study period

**Exclusion criteria**

- Neck pain secondary to vertebral fracture or metastatic cancer
- Complex neck pain (eg, cervical radiculopathy)
- Any professional massage therapy within the last 6 months excluding as part of physical therapy, or visit to the chiropractor or hairdresser
- Potential contraindication to massage (eg, hypersensitivity to touch)
- Hospitalized for psychiatric reasons in the last 3 months
- Occurrence of stroke, transient ischemic attack, heart attack, cervical injury such as whiplash, or hospitalization for chronic obstructive pulmonary disease, emphysema, or congestive heart failure within 6 months of enrollment
- Active suicidal ideation
- Moderate to severe cognitive impairment
- Pending neck surgery
- Involvement in ongoing pain trial or massage study

Eligible Veterans who provided written informed consent were enrolled and underwent a baseline assessment. Randomized care allies also provided written consent to participate at the beginning of the intervention workshop training. Care allies were eligible if their *partnered* Veteran was randomized to the care ally arm, they attended the CA-M training workshop with their Veteran partner and did not have medical concerns that might interfere with giving a massage.

### Recruitment

Primary care providers at the Roudebush Veterans Affairs (VA) Medical Center in Indianapolis, Indiana, and surrounding community-based outpatient clinics were informed of the TOMCATT study details and asked to provide signed approval for the research team to contact their patients for possible study participation. Potential participants were identified by querying the VA’s electronic medical record to create a master list of Veterans meeting the following criteria: (1) neck pain diagnoses per International Classification of Diseases, Ninth Revision codes 721.0 to 723.9 and (2) primary care clinic visits in the past year (a proxy measure of VA care engagement).

A recruitment letter signed by their provider is mailed to qualifying Veterans to describe the study. Letters contain an initial screening for neck pain–related disability that interested Veterans may return if they would like to be contacted to assess their eligibility and possible participation. An appointment is scheduled for eligible Veterans to sign an informed consent statement and the Health Insurance Portability and Accountability Act authorization for those indicating a desire to participate. Baseline interviews and assessments are conducted by a research assistant (MK, EE, or BL) before randomization to minimize ascertainment bias. Veterans also self-identified as being interested in study participation after learning of TOMCATT through acquaintances, word of mouth, or study pamphlets.

### Randomization

Patients were initially assigned to one of the three study arms (TT-M, CA-M, and WL-C) using randomization lists created by the study statistician. Stratified block randomization, with random block sizes of 3 and 6, was used for the original plan to enroll 468 participants (excluding care allies enrolled within the CA-M arm). Sex (male or female) was the only randomization strata used.

### Data Collection Protocol

Table 1 and Table 2 outline the data collection protocol for the Veteran and care ally participants, respectively. Veteran and care ally participants in the CA-M arm also completed a brief learning objectives survey following the training workshop.

**Table 1:** Veteran participant data collection protocol.

Domain	Measure	Items, n	0 months	1 month	3 months	6 months
Demographics	Demographics; disability compensation; comorbidity	36	✓			
Medical comorbidity	Checklist of common medical or psychological conditions		✓			
Neck pain disability	Neck Disability Index [12]	10	✓	✓	✓	✓
Pain severity	Brief Pain Inventory [13]	11	✓	✓	✓	✓
Pain interference	PROMIS^a^-pain [14]	4	✓	✓	✓	✓
Psychological	PHQ^b^-9-depression [15]	9	✓		✓	✓
Psychological	PROMIS-depression [16]	9	✓		✓	✓
Psychological	GAD^c^-7-anxiety [17]	7	✓		✓	✓
Psychological	Veterans Affairs PTSD^d^ screener [18]	4	✓		✓	
Psychological	PTSD-PCL^e^-17 [19]	17	✓		✓	
Psychological	Perceived stress scale [20]	10	✓		✓	
Generic HRQL^f^	MOS-VR^g^-36 [21]	36	✓	✓	✓	✓
Sleep	MOS-Sleep Scale [22]	12	✓		✓	
Somatic	Somatic Symptom Scale-8 [23]	8	✓		✓	✓
Somatic	SSD^h^-12 [24]	12	✓		✓	✓
Pain beliefs	Pain Catastrophizing Scale [25]	10	✓		✓	
Social support	Multidimensional Scale of Perceived Social Support [26]	12	✓		✓	
Treatment satisfaction	Pain-specific satisfaction [27]	3	✓		✓	✓
Intervention credibility	EXPECT^i^ Questionnaire [28]	4	✓		✓	

^a^PROMIS: Patient-Reported Outcomes Measurement Information System.

^b^PHQ: Patient Health Questionnaire.

^c^GAD: General Anxiety Disorder.

^d^PTSD: posttraumatic stress disorder.

^e^PCL: posttraumatic checklist.

^f^HRQL: Health-Related Quality of Life.

^g^MOS-VR: Medical Outcomes Study-Veteran version.

^h^SSD: somatic symptom disorder.

^i^EXPECT: Expectations for Complementary and Alternative Medicine Treatments.

**Table 2:** Care-ally participant data collection protocol.

Domain or measure	Time taken to complete (minutes)	Items, n	0 months	1 month	3 months	6 months
Expectations	1	3	✓		✓	
Brief Pain Inventory [13]	1	3	✓		✓	
PHQ^a^-Stressor Scale	3	9	✓		✓	
PHQ-2-depression	1	2	✓		✓	
GAD^b^-2-anxiety	1	2	✓		✓	
Care ally burden	3	8	✓		✓	

^a^PHQ: Patient Health Questionnaire.

^b^GAD: General Anxiety Disorder.

### Participant Incentives

Veteran participants were reimbursed US $25 per completed outcome assessment, with 4 scheduled assessments (baseline and 1, 3, and 6 months). Participants in the TT-M and WL-C arms were invited to receive all CA-M training and materials after their 6-month interview. Veterans randomized to WL-C are invited to receive a massage from one of the TOMCATT massage therapists. Care allies received a US $50 gift card at the completion of the care ally training and a complimentary massage session.

### Interventions

#### CA-M Intervention

##### Overview

The CA-M intervention consisted of three components: (1) an in-person training workshop led by NM (coinvestigator and licensed massage therapist), (2) an instructional DVD to reinforce the taught concepts, and (3) a printed treatment manual with illustrations and images from workshop materials. Participants were asked to engage in at least three 30-minute CA-M sessions every week at home for the 3-month intervention period. To standardize the delivery and facilitate reproducibility, the content and general structure of the CA-M routine were taught during the workshop. The DVD included a real-time demonstration of the routine for participants to play during applications if desired. Participants were asked to document their massage activities in a study log and return the log sheets monthly.

##### CA-M Routine

The CA-M routine consisted of 13 progressive components ordered to reflect logical seated treatment progression and partially followed the therapist-applied treatment progression from the TT-M arm of the study. Participants were asked to follow the general routine flow and time per area allotments outlined in the protocol and to individualize their massages using the learned techniques. Table 3 displays routine specifics and was included for participants in the treatment manual provided during the workshop.

**Table 3:** The care ally–assisted treatment component, progression, and timing details.

Routine component	Time allotment (minutes)	Component ends at countdown minute	Veteran component activity	Care ally component activity	Accumulated minutes at component’s end
Grounding	1	29:00	Deep breathing and self-grounding and centering	Deep breathing and self-grounding and centering	1
Lymph address	2	27:00	Self-provided lymph drainage	Breathing, grounding, and observing; self-lymph drainage	3
Range of motion	1	26:00	Head, neck, shoulder, and upper back movement	Neck, arms, wrists, hands, and shoulders	4
Check-in or initial connection	1	25:00	Receive and provide feedback	Laying on of hands, making connection, and assessing the tissue with gentle touch	5
Stretching	3	22:00	Receive and apply	Apply to partner and self	8
Warming of neck tissue	2	20:00	Receive and give feedback	Gliding strokes to neck and shoulders	10
More specific neck work	3	17:00	Receive and give feedback	Kneading and point work: neck and shoulders	13
Back work and abdomen	3	14:00	Receive and give feedback and self-apply ab work	Compression, point work, and gliding strokes for upper or lower back	16
Shoulders, neck, and scalp	3	11:00	Receive and give feedback	Apply as continuation of above; add scalp	19
Arms and pecs	3	8:00	Receive and give feedback	Apply to both sides through hands	22
Back, shoulders, neck, and scalp	3	5:00	Receive and give feedback	Final specific work and additional attention items	25
Veteran applied specific point work	4	1:00	Self-apply deep back and front of the neck work	Observe or self-apply	29
Final “sweep” and closure	1	0:00	Receive	Compression, effleurage, gentle tissue movement, or stretching and closure	30

##### CA-M Workshop Specifics

Participant dyads (Veteran and care ally) randomized to CA-M were scheduled to attend a single 3- to 4-hour (including breaks) in-person training workshop held at the Roudebush VA Medical Center. In all, 1-7 dyads (2-14 individuals) attended training workshops. Each training workshop consisted of six parts: (1) introductions and objectives; (2) general instruction (via lecture) on massage, communication approach, safety, CNP, and trigger points that may exacerbate neck pain; (3) massage technique demonstration and supervised practice; (4) specific self-care aspects of the routine and additional and individualized trigger point treatment strategies; (5) demonstration and practice of standardized care ally–assisted massage routine; and (6) questions, closure, and wrap-up.

NM conducted training that demonstrated and encouraged the safe performance of massage tailored to the participants’ needs and abilities. Basic Swedish massage techniques (eg, effleurage [identified as gliding strokes to participants], petrissage [identified as kneading to participants], and compression) were taught during the training, as well as how to use the training DVD and accompanying workbook given to participants. The levels for depth of touch were quantified from very light to so deep that they were not used and described based on tissue-level engagement intention and visual cues from applicant fingertips and recipient skin.

##### CA-M: Instructional DVD Specifics

**Figure 1:**
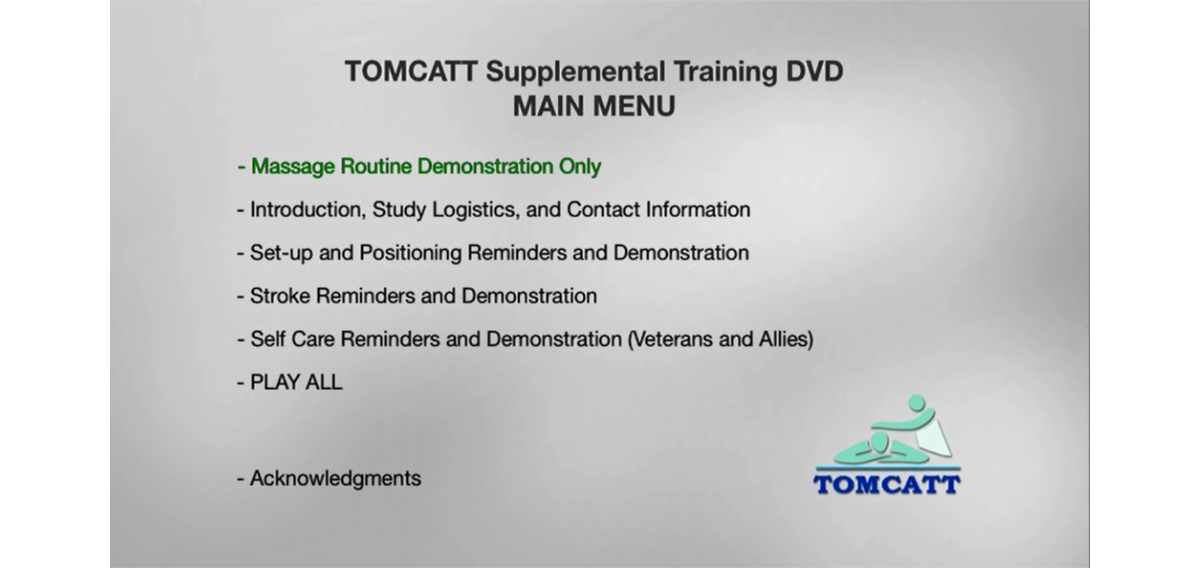
Instructional DVD main menu.

The TOMCATT DVD comprised 5 sections that highlight NM discussing key aspects of the massage routine and workshop-taught techniques. In addition, the DVD included a real-time, full demonstration moving from start to finish through the 30-minute care ally–assisted massage routine. Dyads were encouraged to use the DVD to review technique and positioning instructions and to guide each of the 3 weekly applications of the routine to help with timing and treatment fidelity and to reinforce learning objectives. The DVD main menu (Figure 1) provided viewers with the options to play and view the following choices: (1) the massage routine demonstration only; (2) an introduction from the study principal investigator (MJB), instruction on how to document adherence and compliance to care ally–assisted massage protocol and application, and contact information; (3) instruction and supplemental information or reminders related to how to set up a treatment space within the home, Veteran positioning, and care ally body mechanics during treatment; (4) instruction and supportive reminders regarding massage techniques and how to apply learned techniques to the various body regions (eg, shoulders and arms) addressed in the routine; and (5) review instruction of techniques and concepts learned during the workshop.

Training workshop attendees were also taught how to use the study developed and provided training DVD and an accompanying workbook. The training DVD was a professionally produced, multisectioned DVD designed to complement and reinforce the techniques learned during the training workshop. One section of the DVD was a real-time, full demonstration moving from start to finish through the 30-minute, care ally–assisted massage routine.

##### CA-M: Treatment Manual Specifics 

The CA-M treatment manual is a 32-page, full-color, spiral-bound reference for material taught in the training workshop. The manual content included slides from the training and content arranged to mirror the progression of the training. The manual contained images of associated referral pain patterns, trigger point locations, and treatment of particular trigger points. The treatment manual was given to the participant dyads at the beginning of the CA-M training workshop and had space within it for participants to take notes during the training and use regularly during the study activity as a reference.

##### CA-M: Adherence

Attempts were made to boost adherence and provide posttraining support to participants. Study personnel contacted Veterans and care allies at 2 weeks and in months 2 and 4 to inquire about any barriers to home massage (eg, care ally fatigue or burden, challenges with learning massage techniques and routine, and difficulty adhering to 30-minute sessions). For adherence monitoring, participants were asked to track their use of the DVD and record the time spent on massage on a log form. Monthly log returns were made using postage-paid and addressed envelopes.

#### TT-M Intervention

##### Overview

The TT-M intervention delivery protocol was based on prior research and standardized by treatment intention ordering reflective of typical treatments within therapeutic massage practice for specific pain [29-31]. A Swedish massage–based protocol was chosen because it encompasses the most widely taught and practiced massage techniques that are well-defined procedurally. The allowable techniques included effleurage, petrissage, friction, myofascial, stretching, proprioceptive neuromuscular facilitation, muscle energy technique, stretching, trigger point, compression, rocking, and craniosacral therapy. The specified techniques and activities prohibited by the protocol were deep pressure applied to the anterior neck, movement re-education, shiatsu, and energy work such as Reiki. All massage sessions were delivered in a private treatment room at the medical center.

##### TT-M Protocol

The massage sessions involved a short intake interview followed by 60 minutes of hands-on table time and occurred twice a week (a frequency that balances practicality and efficacy) for 3 months. During the first session, the massage therapist provided an introduction and overview of massage. Thereafter, the TT-M intervention involved a standardized 9-component sequence (Table 4), each with a designated time range that begins with the recipient supine on the massage table. Therapists were instructed to refrain from providing self-care recommendations regarding postures, behavior changes, and sleep.

**Table 4:** The therapist-treated massage protocol details.

Protocol	Time allotment range (minutes)	Description
Range of motion and assessment	3	Hands-on assessment with participant supine on the massage table including active, passive, and resistive range of motion observation and comfort-related dialogue.
Lymph drainage	2-4	Gentle and light touch techniques were applied to the anterior and lateral neck surface, clavicular area, and upper chest and shoulders to encourage lymphatic stimulation and drainage. The techniques mirrored those taught for self-application in the care ally–assisted massage study arm.
Palpation, tissue assessment, and warm-up	1-2	Hands-on gentle palpation, general assessment, and gliding strokes applied to the neck and shoulders were intended to apply massage cream and warm up the tissue.
Specific neck work I	13-22	The massage session progresses to using Swedish massage techniques including stretching applied on participants supine and focused specifically on the base of the skull, neck, shoulders, and upper back (C1-approximately T3) with the intention to address specific muscles and muscle groupings potentially contributing to the pain presentation.
Compensatory patterns and additional concern areas	15-24	Specific work is performed on other areas of the body potentially impacted by or contributing to the participant’s neck pain experience. The participant may change from a supine position to a prone or side-lying position. The arms, back, torso, and legs may all be addressed during this time.
Integration I	7-15	The integration components of the protocol are intended to allow the body an opportunity to incorporate and assimilate tissue changes from the treatment’s specific massage work during the “Specific Neck Work and Compensatory Patterns” components. The recommended and used massage techniques to facilitate work integration included craniosacral techniques; gentle rocking; and long, slow, gliding strokes. The intention here is to also allow the body to “connect” back together once specific areas have had focused attention and other areas perhaps have had little to no attention. Integration components can be applied to participants either prone, supine, or side-lying positions.
Specific neck work II	6-10	A second round of specific neck work near the end of the treatment provides additional time to focus specific massage techniques to the participant’s neck area (as described above). Often times, this component is delivered while participants are in the prone position whereas “Specific Neck Work I” is delivered while participants are in the supine position.
Integration II	2-5	As described above and with the intention to begin the closure process of the treatment. Participant may be asked during this time if there are any additional areas that feel unfinished or would like more work—no new specific work is introduced during this time.
Completion	1-2	This component allows the massage therapist to provide a general closure to the treatment. Often times, clinicians have signature ways in which they may conclude treatment sessions using techniques that include gentle rocking, scalp work, finger-tip brushing, gentle compression, or soft verbal cues. Closure or completion time will often provide a general “signal” to the massage recipient that the session is concluding and allows the end to be expected and not abrupt. The intention here is to support participant relaxation and to provide transition to the posttreatment “world.”

##### Massage Therapists

A total of 9 Indiana state–licensed massage therapists with a range of 3-25 years of experience (median 6 years) were recruited to deliver the massage protocol to participants randomized to the TT-M arm. Therapists were solicited to participate through general and word-of-mouth advertisement approaches and querying professional message organizations.

Study therapists required two types of training: (1) specific study protocols and procedures and (2) VA-required training for privacy and information security.

The study-specific training for protocol and procedures was conducted in person and lasted approximately 3.5 hours. The training was divided into general environment orientation, research processes including duties and responsibilities, and learning specifics related to delivering the massage protocol.

**Figure 2:**
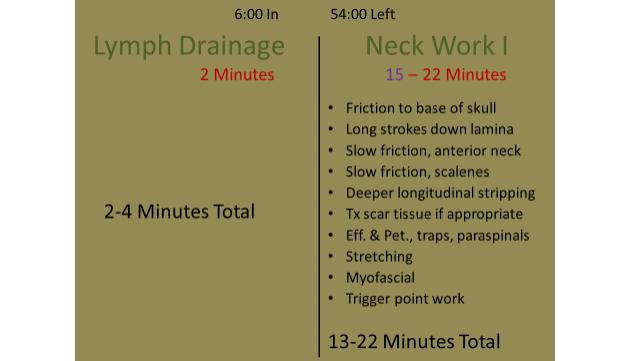
Sample slide from therapist-delivered massage protocol adherence and fidelity PowerPoint. Eff: effleurage; Pet: petrissage; traps: trapezius (upper, middle, lower) Tx: treatment.

The massage therapist protocol adherence and fidelity were addressed in 2 specific ways. First, the therapists launched a timed and silent PowerPoint slide show on a treatment room computer positioned for easy viewing by the therapist during the session. The slide show was timed to advance every 30 seconds through the duration of the 60-minute session and displayed how much time had passed in the protocol, how much time was left in the protocol, the treatment component or components that could occur at that time, and the potential most (in red) and least (in purple) time available to complete any of the possible components (Figure 2). For applicable components, a list of possible techniques and approaches that could be performed during the component was listed as a reminder; therapists were not required to do all or any of the items listed. A silent 15- and 5-second warning (not shown in Figure 2) appeared at the bottom right of the component section to prompt therapist preparation for progress to the next component if needed.

Second, massage therapists completed an electronic fidelity checklist after every session to further support intervention adherence and fidelity. The checklist consisted of 7 sections to ensure that each aspect of the treatment protocol was consistently completed: pretreatment protocol, treatment application protocol, and supported additional notes and documentation. The pretreatment protocol section asked therapists to verify the treatment date, if the participant attended, and that the hands-on, prespecific work aspects of the treatment were completed. The treatment application fidelity section asked therapists to indicate that each treatment protocol component was completed and any specific deviations that occurred. Finally, a note and checklist of allowable general techniques were available at the end of each fidelity checklist to indicate any additional notations.

### WL-C Intervention

Participants in the WL-C arm received check-in calls at months 2 and 4 from the study staff and were administered outcome assessments on the same schedule (baseline and 1, 3, and 6 months) as the treatment groups. Participants in the WL-C were instructed to continue their medical care as normal and to not begin any massage treatment during the 6 months of the study.

### Statistical Considerations

#### Sample Size Justification

The primary contrasts of interest were between the treatment arms (TT-M and CA-M) and WL-C, although analysis of all pairwise comparisons was planned. On the basis of the results from Sherman dosing study [29,31], the change in Neck Disability Index (NDI) at 3 months in the TT-M arm is expected to be significantly better than that in the WL-C arm (effect size=0.8 SD). Greater improvement in the CA-M arm over the WL-C was also expected, assuming a medium effect size (0.5 SD). The initial sample size was determined using a 2-sample independent 2-tailed *t* test. With 396 patients (132 per treatment group), TOMCATT would have 80% power to detect a medium effect size (0.4 SD) among treatment groups in the NDI at the 3-month time point with type I error set at 0.017 (0.05/3) to maintain familywise error at 0.05. An approximate 15% dropout rate at 3 months was expected, and it was initially planned to enroll 468 patients (156 per treatment group).

#### Statistical Analysis

##### Overview

Balanced baseline characteristics of the study participants is expected across the 3 treatment groups due to randomization. Baseline demographic and clinical characteristics will be compared among the treatment groups using the appropriate tests. Variables found to be significantly different will be included in the subsequent regression models.

##### Main Analysis (Aim 1) of the Primary Outcome (NDI Total Score)

All outcomes are collected at baseline and 1, 3, and 6 months. For the primary outcome of the NDI total score, a linear mixed effect model will be used with an appropriate covariance structure to compare each treatment arm to the WL-C arm on change scores at the 3-month time point. Fixed effects will include treatment, time (as categorical), and treatment by time interaction. Differences in change scores among groups at other time points (1 and 6 months) will also be reported. Associations among repeated measures within participants will be examined, and a data-driven approach will be used to determine the appropriate variance-covariance structure [32]. The Šídák method will be used to adjust for multiple comparisons. Type I error will be set at 0.017 for the primary comparisons to maintain the familywise error at 0.05. An intent-to-treat approach is planned with the primary end point at 3 months and evaluation of “early” response at 1 month and “sustained” response at 6 months after randomization.

##### Analysis of Secondary Outcomes (Aim 2)

TOMCATT is not specifically powered for secondary outcomes, and a cautious interpretation of the secondary analysis results is planned. For pain intensity, those with a clinically relevant >30% reduction from baseline will be reported as “responders.” For the NDI, a “responder” will be defined as a decrease of >5 points from baseline to 1, 3, and 6 months for each participant. A generalized linear mixed model with predictors of group, time, and their interaction will be used. The model will also include a random subject effect to accommodate the potential correlation among observations from the same participant [33]. The primary contrast of interest will be the difference in proportions at 3 months between each treatment and the WL-C group. Similar regression modeling strategies will be used to assess the exploratory outcomes of pain coping, sleep problems, satisfaction, and social support.

##### Moderator Analyses

Baseline anxiety (General Anxiety Disorder-7) and depression (Patient Health Questionnaire-9) will be tested as potential moderators of the primary outcome (NDI), as well as secondary outcomes, as secondary analysis. Testing these measures as moderators will provide insight into the generalizability of the interventions. For example, if the intervention loses effect in patients with high anxiety or depression, they may not be suited to the intervention.

##### Missing Data

Missing data in 2 different forms are expected: missing data by attrition and intermittent missing of observations. Attrition of <15% was expected at the 3-month follow-up. Differential attrition among study arms was expected during study planning or initiation, yet the potential that care ally burden could lead to more attrition in the CA-M arm and transportation barriers could lead to more attrition in the TT-M arm was acknowledged by the study team. The effects of missing observations due to attrition will be examined by analyzing the patient characteristics associated with dropout. Multiple imputation techniques may be used to alleviate the impact of missing data. However, if the pattern of missing data is nonignorable, more complex modeling approaches to model the missing data may be used. Finally, sensitivity analyses will be conducted to ensure the validity of study findings.

## Results

### Overview

The TOMCATT pretrial activities were launched in July 2016 and included massage therapist recruitment, training, and onboarding; the finalization of training materials; data collection documents and databases created; and research personnel hired and trained.

**Figure 3:**
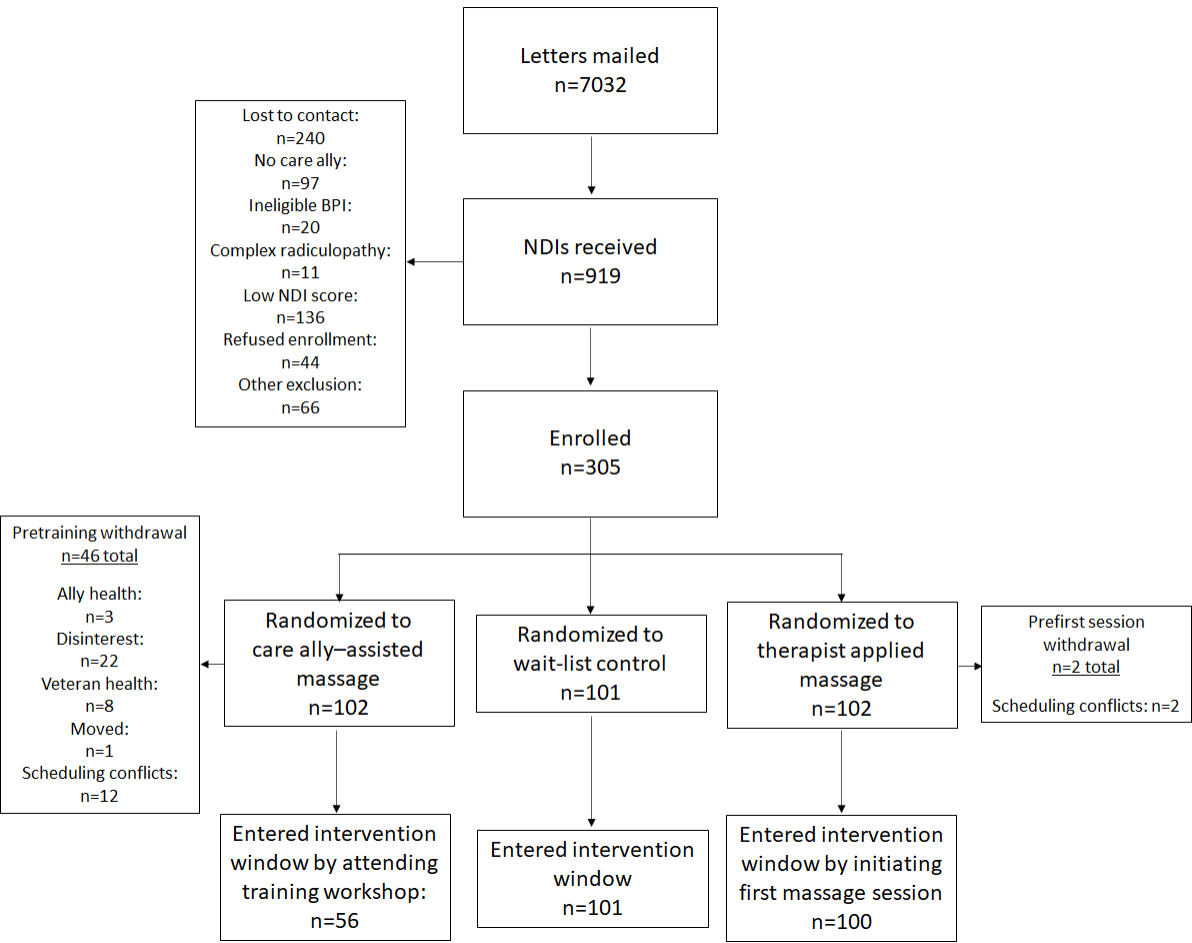
Premodification recruitment, randomization, and intervention initiation flow diagram. BPI: Brief Pain Inventory; NDI: Neck Disability Index.

Participant recruitment began in May 2017. Figure 3 depicts the results of screening, eligibility determination, enrollment, randomization, and treatment initiation for the first 30 months of TOMCATT. Potential participants were identified through the electronic medical record as having CNP, of whom 7032 were sent a study invitation letter that included a screening NDI form for interested individuals to return to learn more and be further screened for enrollment eligibility. Various additional recruitment methods have been used, but their yield is unclear. The NDI screening forms yielded 33.2% (305/919) of the enrolled participants. In all, 14.8% (136/919) of the received NDI form scores did not meet the neck pain with disability thresholds required to participate, whereas another 26.1% (240/919) of potentially eligible participants returned NDI forms but could not be contacted.

### Participant Recruitment, Enrollment, Intervention Initiation, and Retention

Randomization resulted in 102, 102, and 101 participants in the CA-M, TT-M, and WL-C arms, respectively, in the first 30 months. All WL-C participants progressed to the 12-week nonintervention phase of the study, with 93.1% (94/101) completing an outcome assessment (data not shown). In all, 98% (100/102) of participants in the TT-M initiated the intervention. Of the 102 participants, only 2 (2%) failed to schedule and attend their first TT-M treatment and withdrew from the study owing to schedule conflicts.

For CA-M, only 54.9% (56/102) of the randomized patients attended training and initiated treatment. Disinterest was the primary reason (22/46, 48%) indicated for noninitiation, followed by scheduling issues (12/46, 26%). Among those who attended training, an average of 42 days passed between baseline data collection and training or treatment initiation. Participant engagement was further challenged by inconsistent treatment log-return compliance, despite most participants (52/56, 93%) completing one or more follow-up outcome assessments (data not shown). Nearly 45% (25/56) of participant dyads returned no compliance logs, whereas nearly one-third of the participants (17/56) returned logs for each of the 12 intervention weeks. A majority of CA-M dyads (31/56, 55%) returned at least one week of compliance logs.

### Efforts to Improve CA-M Arm Treatment Initiation

Several mitigatory steps were taken to improve treatment initiation in the CA-M arm over approximately 14 months. To accommodate individual schedules, training workshops were scheduled 3 times per month, which included at least one Saturday, a morning training session during the week, and an afternoon training session during the week per month; any of which was held for as long as at least one dyad attended. Several additional training workshops were scheduled per individual dyad scheduling needs.

In an effort to retain participants in the CA-M arm and facilitate treatment initiation for those with care ally barriers, a matching approach was launched whereby former Veteran participants who expressed willingness were matched with enrollees who ultimately did not have a care ally willing or able to attend training and provide care ally–assisted intervention for 12 weeks. An enrollee with care ally participation barriers agreed to be matched but did not attend the scheduled training, despite the matched, stand-in care ally attending.

Although not initiated, a modified training approach was developed for CA-M arm participants as an alternative to the 3.5-hour training seminar. The modified approach was composed of a combination of at-home learning and supportive applied laboratory experiences. The developed approach included participants accessing digitally recorded didactic information and materials from a supportive DVD on their own time in a structured and prompted format. Once complete, the dyad would schedule a 1-hour hands-on application session with NM to reinforce content and ensure safe and appropriate application of the intervention techniques.

### Modified Trial Protocol

#### Overview

The decision to modify the TOMCATT design was informed by disproportionate attrition before treatment initiation and poor adherence to CA-M treatment logs. These challenges persisted, despite several procedural modifications to support participation and adherence. The modified study design to remove the CA-M arm was developed and approved by the study funder and institutional review board in November 2019 and December 2019, respectively. Participants enrolled and randomized to the study before the postmodification date progressed through study completion based on their original group assignment.

#### Modified Eligibility Criteria, Recruitment, Enrollment, and Randomization

The inclusion criterion for participants to have an identified care ally was removed from the modified study design. The recruitment and enrollment procedures remained the same. After modification to a 2-arm study, a new set of randomization lists was created. Within each strata defined by sex, patients were randomized 1:1 to the TT-M or WL-C group using block sizes of 4.

#### Modified Design Sample Size Justification

Because of high attrition in CA-M (62/98, 63%), TOMCATT was modified into a 2-arm study (TT-M and WL-C). The modified study will focus on comparison of the TT-M and WL-C. At modification, it was assessed that 100 patients per group (N=200) would provide 80% power to detect a 0.4 SD in NDI change from baseline between TT-M and WL-C with type I error of 0.05. The premodification attrition rate at 3 months in the TT-M and WL-C groups was 23.9% (47/197). Thus, the enrollment of 264 (200/0.76) or 132 per group was planned during the postmodification period. The comparison of CA-M to WL-C obtained before the study modification will be considered a secondary analysis and reported in a subsequent manuscript.

#### Modified Design Statistical Analysis

The analysis plan for the modified study will be conducted in a manner similar to that of the initial 3-arm randomized controlled trial. Randomization is expected to achieve balanced baseline characteristics of study participants among the study groups. Primary (NDI) and continuous secondary outcomes (pain intensity, depression, anxiety, and pain cognition) will be assessed using a linear mixed model approach. Type I error will be set at 0.05. The Šídák method will be used to adjust for multiple comparisons at a given time point for the secondary outcomes. Noncontinuous outcomes will be assessed using a generalized linear mixed model. In the revised design, missing data from attrition and intermittent missing observations are expected. Attrition of approximately 24% is expected by the 3-month follow-up and has been accounted for in the modified sample size calculation. The effects of missing observations due to sample attrition will be examined based on the patient characteristics associated with early dropout.

### Modified TOMCATT Design Progress Through the Initiation of the COVID-19 Pause

**Figure 4:**
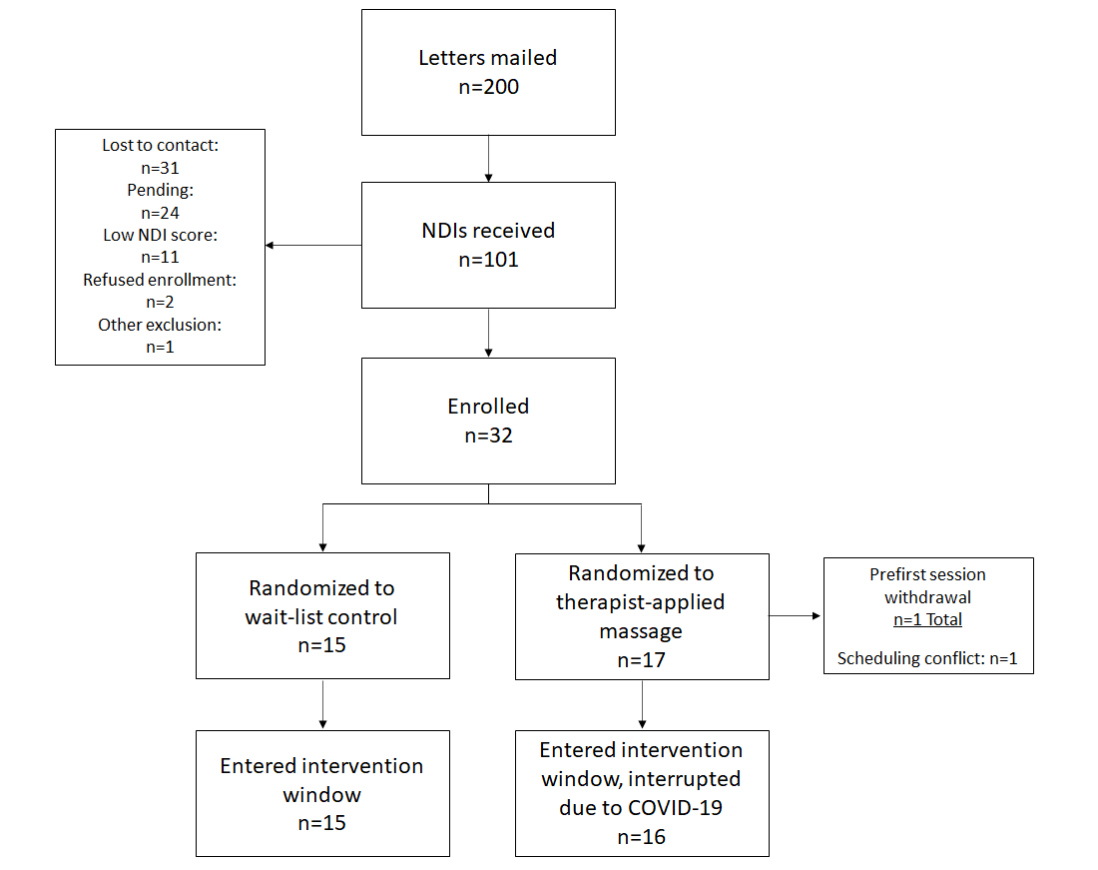
Postmodification recruitment, randomization, and intervention initiation flow diagram. NDI: Neck Disability Index.

Figure 4 displays the flow diagram for recruitment, enrollment, and treatment initiation from November 2019 to March 2020, when the COVID-19 pause began. Recruitment efforts resulted in 50% of the recalculated needed enrollment achieved in just over 4 months. Of those enrolled, all but one TT-M participant initiated treatment (TT-M).

## Discussion

### Principal Findings

The TOMCATT trial was initially designed to test the effectiveness of 2 different massage interventions versus WL-C. Treatment engagement and retention challenges have emerged and raised questions about the feasibility of CA-M. Consequently, the study design was modified, resulting in a simpler, 2-arm study. This decision, although difficult, allowed the TOMCATT study team to focus and redirect recruitment efforts on the 2 remaining study arms. Promising recruitment and retention success for the postmodification efforts point to promising expectations for TOMCATT’s resumption efforts from the COVID-19 pause.

### Comparison With Prior Work

Caregiver-delivered massage has been shown to be feasible and effective in 2 previous Veteran-focused studies [10,11], but such an approach has not been studied for a chronic musculoskeletal pain population. The treatment engagement and retention challenges that disproportionately affected the CA-M arm were greater than expected, based on previously published studies [10,11]. It is speculated that these challenges have emerged for several reasons. First, participants randomized to the CA-M group may have been disappointed that they were not randomized to the TT-M group given the popularity of massage. It is well known that participants have strong preferences for treatment allocation within trials [34], and these preferences can impact follow-up rates, attrition, and treatment outcomes [35]. Second, dyadic research poses unique challenges in recruitment, retention, attrition, data collection, and analysis [36] relative to nondyadic research. Studies of chronic pain may heighten the challenges of dyadic research, because chronic pain can have a significant impact on a person’s social relationships by triggering symptoms of anxiety, depression, and anger [37]. Third, CA-M was an intensive intervention that involved attending a one-time, multihour workshop; a DVD for regular home use; a treatment session log to complete weekly; and delivery of 30-minute massage sessions by the care ally 3 times a week for 12 weeks. The substantial time investment and significant requirement for active dyadic participation may have been too great for some participants who never engaged in the intervention or withdrew from the study.

The qualitative interview data collected in a subset of TOMCATT participants are anticipated to elucidate some challenges that emerged within the original design and may provide potential insights into ways to improve treatment engagement, retention, and interpret eventual trial results. In these interviews, the participants are asked about their prerandomization arm preferences, treatment perceptions, and outcome expectations. Qualitative data collection and analysis may highlight a desire to incorporate treatment preferences into the design of future massage trials. Furthermore, assessing the sociodemographic and clinical correlates of treatment engagement and retention, especially among CA-M participants, are planned.

### Limitations

This manuscript reports only preliminary findings related to enrollment and intervention uptake to explain the modification rationale from a 3-arm to a 2-arm study design and to describe the modification methodology. Furthermore, the COVID-19 pandemic has caused significant disruptions to TOMCATT, especially the delivery of TT-M. All in-person noncritical research activities were suspended in March 2020. Thus, all in-person TOMCATT activities, including delivery of massages, were forced to halt owing to safety concerns and institutional mandates. Because of intermittent surges in COVID-19 infection rates throughout 2020 and early 2021, TOMCATT continued to suspend in-person research activities owing to safety concerns for participants, massage therapists, and study staff. As a result, recruitment and massage treatment delivery for TOMCATT did not restart until June 2021, after infection rates had declined and safety mitigation factors were in place and believed to be effective. Study recruitment and enrollment began gradually with study capacity reached and a smooth process implemented by December 2021.

### Conclusions

Although the use of informal caregivers to provide massage is an innovative care delivery model, it was not feasible in our study of Veterans with CNP, which was hampered by low engagement and high attrition. TOMCATT’s continued efforts with the modified study design will provide a better understanding of the feasibility and effectiveness of an on-site therapist-applied massage treatment model for Veterans with CNP, an approach that demonstrates feasibility and acceptability. The incorporation of patient preferences into the study design is planned for future trials to improve engagement and retention in all care approaches.

## Abbreviations

CA-M: care ally–assisted massage
CNP: chronic neck pain
NDI: Neck Disability Index
TOMCATT: Trial Outcomes for Massage: Care Ally–Assisted Versus Therapist-Treated
TT-M: therapist-treated massage
VA: Veterans Affairs
WL-C: wait-list control group

## Data Availability

The data set supporting this article is not available. According to Department of Veterans Affairs policy, these data are stored behind the Veterans Affairs firewall and cannot be shared even after deidentification. Investigators interested in analyses of the existing data are encouraged to contact the corresponding author.
